# Exploring behavioural factors influencing COVID-19-specific infection prevention and control measures in Finland: a mixed-methods study, December 2020 to March 2021

**DOI:** 10.2807/1560-7917.ES.2022.27.40.2100915

**Published:** 2022-10-06

**Authors:** Anna-Leena Lohiniva, Saija Toura, Dinah Arifulla, Jukka Ollgren, Outi Lyytikäinen

**Affiliations:** 1 The Finnish Institute for Health and Welfare, Helsinki, Finland

**Keywords:** Infection prevention, COVID-19, long-term care facilities, behaviour change

## Abstract

**Background:**

Compliance with infection prevention and control (IPC) measures is critical to preventing COVID-19 transmission in healthcare settings.

**Aim:**

To identify and explain factors influencing compliance with COVID-19-specific IPC measures among healthcare workers (HCWs) in long-term care facilities (LTCF) in Finland.

**Methods:**

The study included a web-based survey and qualitative study based on the Theoretical Domains Framework (TDF). The link to the anonymous survey was distributed via email to LTCFs through regional IPC experts in December 2020. Outcome was modelled using ordinary logistic regression and penalised ridge logistic regression using regrouped explanatory variables and an original, more correlated set of explanatory variables, respectively. In-depth interviews were conducted among survey participants who volunteered during January–March 2021. Data were analysed thematically using qualitative data analysis software (NVIVO12).

**Results:**

A total of 422 HCWs from 17/20 regions responded to the survey. Three TDF domains were identified that negatively influenced IPC compliance: environmental context and resources, reinforcement and beliefs about capabilities. Twenty HCWs participated in interviews, which resulted in identification of several themes: changes in professional duties and lack of staff planning for emergencies (domain: environmental context and resources); management culture and physical absence of management (domain: reinforcement), knowledge of applying IPC measures, nature of tasks and infrastructure that supports implementation (domain: beliefs about capabilities), that explained how the domains negatively influenced their IPC behaviour.

**Conclusions:**

This study provides insights into behavioural domains that can be used in developing evidence-based behaviour change interventions to support HCW compliance with pandemic-specific IPC measures in LTCFs.

**Public Health impact of this article**

**What did you want to address in this study?**

Infection prevention and control (IPC) measures are an important means to prevent transmission of infectious diseases in long-term care facilities (LTCFs). We wished to identify factors that influence the compliance with COVID-19 pandemic-specific IPC measures of nursing staff in LTCFs in Finland.

**What have we learnt from this study?**

From the participants' answers to the questionnaires and interviews, we find COVID-19-specific IPC measures were influenced by staffing, management, knowledge and infrastructural factors. Interventions to encourage the implementation of IPC measures must include different elements to address the factors that influence the compliance with the measures.

**What are the implications of your findings for public health?**

As factors influencing IPC measures are context-specific, they must be understood to be able to develop appropriate interventions to encourage uptake of the measures.

## Introduction

Long-term care facility (LTCF) residents have been among the populations at greatest risk of morbidity and mortality related to coronavirus disease 2019 (COVID-19) [1,2,3]. Although Finland (5.5 million population) was able to minimise the impact of the pandemic with only ca 1,000 reported COVID-19-related deaths up to June 2021, a notable proportion of deaths (40%) occurred among LTCF residents [4], similar to the situation in many European countries [5].

In healthcare settings, such as LTCFs where contacts between healthcare workers (HCWs) and residents are frequent and close, infection prevention and control (IPC) measures are essential to mitigate the spread of infectious diseases [6,7,8]. Throughout the COVID-19 pandemic, both public and private LTCFs in Finland have received IPC guidelines for hand hygiene, use of personal protective equipment (PPE) and environmental cleaning from various sources at national and local levels; these include the Ministry of Social Affairs and Health and the Finnish Institute for Health and Welfare, which guides and supports municipalities, as well as healthcare districts and regional state administrative agencies in their work to prevent infectious diseases. LTCFs also guidance to reorganise their group activities and dining to minimise physical contact. At the beginning of the pandemic (March–April 2020), visitors to LTCFs were prohibited, with the exception of those visiting residents who were in critical or terminal condition. As the pandemic continued from May 2020 onwards, the guidance allowed more flexibility to ensure better well-being of the residents of LTCFs [9].

Compliance with these new measures required behaviour modifications by HCWs of LTCFs. Behaviour change theories highlight that to modify or change HCW behaviour, there is a need to understand the factors that driving compliance [10]. However, little is known about the barriers and facilitators related to nursing staff compliance with COVID-19-specific IPC measures in Finland or elsewhere [6,11,12]. A recent review provides some evidence about factors driving IPC behaviours during the pandemic including knowledge of guidelines, training to use PPE, management support, infrastructure and provision of PPEs, fear of infection, peer support and fear of being stigmatised [6]. Several studies indicate that behaviour change in HCWs, including changing IPC practices, is a complex phenomenon influenced by numerous factors, including social and cultural issues, that must be understood in each context to change them [13,14]. Other studies underscore that behavioural change is more effective if interventions are based on theories of behavioural change [6,15].

To better understand how to optimise the application of IPC measures during the pandemic, we conducted a survey and qualitative study on HCWs to identify and explain factors that influence the implementation of IPC measures in LTCFs. The findings can be used to propose interventions and policies to better support HCW compliance with IPC measures during future epidemics in Finland and other similar settings.

## Methods

### Study design

This is a mixed-methods study including a survey followed by a qualitative study. Both were based on Theoretical Domains Framework (TDF). TDF is a framework consisting of 14 domains of behaviour including cognitive, affective, social and environmental determinants and influences on behaviour [13-15]. The survey questions were developed by expert consensus. The process was initiated by defining each TDF domain to match IPC practices, followed by listing items in each domain and identifying those most appropriate for the survey. The selected items were formulated into questions and piloted. The questions for the qualitative study were developed to cover all TDF domains. They included open-ended questions with a set of probing questions. TDF domains and related survey questions are shown in Table 1.

**Table 1:** Theoretical domain framework and related survey questions for healthcare workers of long-term care facilities to identify factors influencing compliance with IPC measures during the pandemic, Finland, December 2020–March 2021

Domains (n = 14)	Survey questions (n = 17)
Knowledge	How do you evaluate your COVID-19-specific IPC information?
Skills	How do you evaluate your professional skills to care for COVID-19 patients?
Social influences	How do think your social relations (outside of your workplace) influence your compliance with COVID-19-specific IPC measures?
Social and professional role and identity	How do you evaluate your roles and responsibilities at work and their influence on your ability to comply with COVID1–9-specific IPC measures?
	How do you evaluate your social relations at the workplace and their influence on your ability to comply with COVID-19-specific measures?
Reinforcement	How do you evaluate management influences on your ability to comply with COVID-19-specific IPC measures?
	How do you evaluate feedback from management as influencing your compliance with COVID-19-specific IPC measures?
Beliefs in capabilities	How do you evaluate your ability to improve your compliance with COVID-19-specific ICP measures?
Beliefs in consequences	How do you evaluate lack of COVID-19-specific IPC compliance as negatively influencing patient care?
Behavioural regulation	Do you have concrete plans to improve your current COVID-19-specific IPC measures?
Environmental context and resources	How sufficient is staffing to be able to comply with COVID-19-specific IPC measures?
Memory, attention and decision processes	How frequently do you forget to comply with COVID-19-specific IPC measures?
	How do you evaluate the impact of compliance with COVID-19-specific IPC measures on your psychological well-being?
Emotions	How do you evaluate the amount of strong feelings linked with compliance with COVID-19-specific IPC measures generates (fear, sorrow etc.)?
Goal	Have you set a personal goal for yourself to improve compliance with COVID-19-specific IPC measures?
Intention	Have you made a conscious plan to improve compliance with COVID-19-specific IPC measures?
Optimism	Do you believe that you can contribute to reducing the risk of transmission by using IPC measures?

COVID-19: coronavirus disease; IPC: infection prevention control.

### Survey

The survey data were collected using a web-based questionnaire. All HCWs including nursing staff, doctors and others providing care in LTCFs were eligible to join the study. Recruitment for the survey took place through one regional infectious disease doctor in each region (n = 20) who was responsible for communicable disease control in the healthcare district. They were requested to share the survey link via email with the management of LTCFs in their healthcare districts who would then invite the HCWs to participate in the survey. The survey was open for one month during December 2020. Respondents participated anonymously but they could also provide their contact information to participate in an in-depth interview.

In addition to answers to the TDF domains-related questions (Table 1, n = 17), the survey questionnaire included questions about background information (n = 5: healthcare district, municipality, type of facility, owner of the facility, profession) as well as questions of IPC guidelines (n = 2: availability, source) and problem areas in compliance with the guidelines (n = 4: hand hygiene, PPE, environmental cleaning, other). The analysis was based on both ordinary logistic regression and penalised ridge logistic regression using regrouped explanatory variables and original, more correlated set of explanatory variables, respectively. We performed a forward selection of variables using Akaike information criteria (AIC) when evaluating the best selection of variables for the final model. The outcome was identifying problems in complying with the guidelines, and explanatory variables were based on replies to TDF domains-related questions as well as the type of profession.

### Qualitative study

The qualitative study comprised in-depth interviews with HCWs in LTCFs who provided direct care for residents. Staff members who only had managerial duties were excluded. Those who submitted their contact information after participating in the web-based survey were contacted by a research team member to schedule a telephone-based interview, which was organised based on the availability of the interviewees.

The interviews were conducted between January–March 2021 by the researcher (ALL), who used a question guide that consists of open ended and semi-structured questions (Supplementary file 1: Question guide for qualitative interviews) and is trained and experienced in qualitative data collection and analysis methods. The interviews lasted 20–40 min. All respondents gave verbal consent to participate. The researcher took notes during the interview and expanded on them directly after each interview to minimise recall bias. All data were confidential and no identifiers were recorded.

The analysis of each interview was based on a thematic analysis, which was conducted by the researcher (ALL). The analysis followed the TDF [13] that included identifying codes and categories within all 14 domains of the framework. The process started with a data familiarisation process during which the analyst read the interview notes several times to get an overall idea of the dataset and to create an initial set of codes. During the coding, emerging new codes were included, followed by refining, expanding codes and developing categories [16]. NVIVO12 was used in the coding process. The initial analysis was shared with the study team (ST, OL) to get a consensus on the emerging categories. In the final stage, the analyst (ALL) developed the interpretation. The syntheses of the results served as the foundation for operational recommendations to improve healthcare workers’ compliance with IPC measures. The final set of codes, categories, and themes is displayed in a codebook (Supplementary file 2: codebook of TDF domains).

## Results

### Survey responses

A total of 422 HCWs from 17/20 healthcare districts responded to the survey. Many (171/422, 40.5%) respondents were from the healthcare district around the capital city of Helsinki and most of them (342/420, 81.4%) worked in nursing homes (Table 2). Approximately half of the respondents worked in the public sector and the other half in the private sector. Almost half of the respondents (n=192/421, 45.6%) were assistant nurses and one-third were registered nurses (135/421, 32.1%). Approximately one-fifth of the respondents (91/421, 21.6%) were other staff members that included managers, therapists, counsellors, and social workers. Less than 1% (3/422) of the respondents were physicians.

**Table 2:** Background information of survey participants working in long-term care facilities, Finland, December 2020–March 2021 (n = 422)

Variable	n	%
Type of facility		
Healthcare centre ward	15	3.6
Nursing home (supervision 24 h/day)	342	81.4
Other^a^	63	15.0
Owner of the facility		
Public	236	56.1
Private	185	43.9
Profession		
Physician	3	0.7
Nurse	135	32.1
Assistant nurse	192	45.6
Other^b^	91	21.6

^a^ Another unspecified type of health care facility.

^b^ Managers, therapists, counsellors and social workers.

During the pandemic, the respondents followed the IPC guidelines from the healthcare district (38.5%, 162/421), the Finnish Institute for Health and Welfare (24.5%, 103/421), the municipality (20.2%, 85/421), their own facility (14.3%, 60/421) or other (1.9% 8/421). Only 0.7% (3/421) of the respondents had not followed any kind of COVID-19-specific IPC guidelines. Most respondents (83.2%, 351/422) had experienced difficulties in complying with at least one COVID-19-specific IPC measure (hand hygiene, PPE, environmental cleaning, other). Over one-third (36.5%, 154/422) of the respondents found it difficult to use PPE and approximately one-quarter (25.6%, 108/422) experienced challenges with environmental cleaning. Only 3.6% (15/422) of the respondents reported having difficulties with hand hygiene procedures. Of the respondents, 29.1% (123/422) reported other issues, most commonly related to visiting policies (33.6%, 40/119).

Three TDF domains were identified as influencing compliance with COVID-19-specific IPC measures (Supplementary file 3: logistic regression). Environmental context and resources were significantly associated with IPC compliance by two methods, the logistic regression (odds ratio (OR): 0.55; 95% confidence interval (CI): 0.32–0.94; p = 0.027) and ridge regression (OR: 0.52; 90% CI: 0.33–0.82; p = 0.018). Per the ridge regression, there was a similar association related to reinforcement and beliefs about capabilities (OR: 1.73; 90% CI: 1.00–2.99; p = 0.10).

### Qualitative study

Of the total 422 HCWs, 50 respondents initially agreed to participate in an interview. However, during scheduling, only 20 were available for an interview. Respondents included 14 nurses and six assistant nurses. Approximately half (11/20) worked in the public governmental sector and the remainder in the private sector. All respondents were female, and they came from (11/20) healthcare districts, but mostly from the capital area.

The findings provide insights into three TDF domains (environmental context and resources, reinforcement and beliefs in capabilities), which were identified in the survey as influencing compliance with IPC behaviours.

#### Domain: Environmental context and resources

Respondents were asked to explain how insufficient staffing – a factor linked with the TDF domain environmental context and resources – influenced their compliance with COVID-19-specific IPC measures during the pandemic. Two themes merged from the discussion: changes in professional duties and lack of staff planning for emergencies.

##### Changes in professional duties

Most respondents explained that many COVID-19-related IPC procedures were labour intensive such as bringing residents to the dining room in multiple small groups or organising meeting appointments for the families and friends of the residents. Accordingly, there was less time to manage work routines including IPC measures.

“*I found myself running up and down between the dining room and the resident rooms numerous times as we provide lunch for an extended period to manage physical distancing. To be honest, IPC is not on my mind.*” (Assistant nurse, private sector)

Some respondents explained that their facilities lacked a sufficient workforce before the pandemic, which was further exacerbated during the pandemic, resulting in less time to provide patient care and give attention to hygiene measures.

“*We always had lack of staff but now during the pandemic the situation is turning to be impossible. I feel bad not being able to provide real care as all my energy goes to manage the daily routine. Everything gets difficult to implement, not just IPC measures.*” (Assistant nurse, private sector)

##### Lack of staff planning for emergencies

Most of the respondents explained there was not a new staffing plan during the pandemic that took into consideration the changes in professional duties. Some respondents explained that when they had COVID-19 cases among residents, adjustments were made to minimise exposure by having dedicated staff take care of them. However, such changes often overloaded the rest of the staff and reduced their opportunities to ensure IPC measures were fully implemented.

“*The times that we had coronavirus cases in our ward we had to work really hard. Surely, we would sometimes skip some IPC measures. It was not intentional. It can just happen when everyone is overly busy.*” (Assistant nurse, public sector)

A few respondents from the private sector explained that their workplace had made staffing adjustments that considered potential changes in duties and COVID-19 cases, but these plans included short-term hired nursing staff who were not always aware of COVID-19-specific IPC measures.

“*It is really difficult with these hired folks because they don’t know IPC measures and we have many more to follow during the pandemic.*” (Nurse, private sector)

#### Domain: Reinforcement

Respondents were asked to explain how management behaviour and attitudes, and management feedback linked with the TDF domain reinforcement influenced their compliance with COVID-19-specific IPC measures. Two central themes were identified: management culture and the physical absence of management.

##### Management culture

Most respondents explained that positive feedback from the management was a sign of appreciation. However, many respondents, particularly those from the public sector, pointed out a lack of systematic feedback. One nurse highlighted that they only received negative feedback. Several respondents from both the public sector and private sector highlighted that supervisors’ rounds to observe their IPC procedures were usually done on an ad hoc basis and, during the pandemic, this practice was basically non-existent.

“*[The management] are not used to giving any feedback. This is the system in our workplace.*” (Assistant nurse, public sector)

A couple of respondents from the private sector explained having had the opportunity to engage in IPC-related discussions with their supervisors during the pandemic as their management culture was highly participatory.

“*Our management engages us in informal discussions about our new IPC measures. It helps to apply to these measures and it gives us an opportunity to get their feedback.*” (Nurse, private sector)

Two respondents from small LTCFs explained that all staff, including their supervisors, were engaged in patient care and therefore feedback and comments from the supervisors did not feel natural.

“*We are a small house. We all work together. I would not feel comfortable observing my colleagues.*” (Nurse, private sector)

Two respondents were designated specifically as ‘corona help’ that included working in facilities with COVID-19 patients. Both highlighted that they had not received any supervision or feedback from any of the wards where they had assisted for a duration of a few days and up to 6 weeks.

“*Nobody really pays attention to extra workforce like me. Supervisors don’t observe if I use a mask or anything else. It is as if they do not consider me as their staff*.” (Nurse, public sector)

##### The physical absence of management

Several respondents from the private sector explained that during the pandemic their management worked from home and accordingly there was no direct supervision or feedback.

“*We haven’t seen our supervisor for a very long time. They have worked from home since the beginning of the pandemic.*” (Nurse, private sector)

One respondent from the private sector disagreed with this view, claiming that supervisor presence was not necessary to ensure compliance with IPC measures as long as the supervisor was respected.

“*We have a supervisor who does not give us much direct feedback. But it is fine. We follow all her instructions. She is highly respected*.” (Assistant nurse, private sector)

Some respondents from the public sector explained that their supervisors were usually physically present, but they stayed in their office. Some respondents also pointed out that social coffee breaks were less frequent during the pandemic. Although such casual social engagements were not dedicated times for feedback on IPC procedures, they were opportunities to ask questions to supervisors.

“*We have seen less of our supervisors during the pandemic. You are pretty much on your own.*” (Assistant nurse, public sector)

#### Domain: Beliefs in capabilities

Respondents were asked to explain why and how beliefs in capabilities influenced their compliance with COVID-19-specific IPC measures during the pandemic. Three themes emerged to explain this: knowledge of applying IPC measures, the nature of the tasks and the infrastructural support.

##### Knowledge of applying IPC measures 

The majority of respondents explained that sufficient knowledge meant that they received information that was understandable, applicable in their context, and available continuously. However, many respondents had the opposite experience during the pandemic, and received too much information, too generic information, and instructions that were too difficult to apply in reality.

“*When we get information all the time and it is changing all the time, I get a feeling that I am not in control. I will never be able to know everything*.” (Nurse, private sector)

A few respondents evaluated themselves as knowledgeable based on their long working experience (≥ 10 years) or on the fact that they knew how to obtain information if needed. For example, one respondent explained getting in touch with the regional IPC focal point whenever she was unsure of certain IPC procedures, whereas another respondent highlights that it was easy to get advice from the regional infectious disease doctor. 

“*It required some courage to get in touch with experts but once I did, I noticed that it is an easy and an excellent way to learn. This is one of the positive things that happened during the pandemic.*” (Nurse, private sector)

##### The nature of the tasks

Many respondents highlighted that compliance with some of the IPC measures was challenging such as keeping physical distance between residents that had memory loss as well as between themselves and the other residents.

“*Keeping physical distance is absolutely impossible. We cannot lock people in their rooms, and they keep forgetting all the time*.” (Nurse, public sector)

Many respondents also highlighted that communicating with residents’ family members about new visiting times and hours was a real challenge that the HCWs often did not manage well. Some respondents explained that such discussions touched on residents’ rights and accordingly were very sensitive. Other respondents also pointed out that they were not authorised to forbid family members from visiting, which made communication about the new visiting rules difficult.

“*I don’t always easily manage discussions with the family members. They refer to their rights and make our requests to abide by our new visiting rules really difficult*.” (Assistant nurse, private sector)

##### Infrastructural support

Some respondents mentioned that the use of protective barriers at work was difficult because they did not have any dedicated changing rooms or staff rooms. One respondent explained that their wards were in an old building that had only a few hand-washing areas and this made intensified hand hygiene procedures a challenge.

“*Our workplace is homelike, so it is very unpractical because we as staff members do not have any space for ourselves. When you change your mask or gloves you have residents around all the time.*” (Nurse, private sector)

A few respondents explained that the use of masks had been difficult for them at the beginning of the pandemic because masks created allergic reactions. The problem was usually solved by trying different types of masks until the right one was identified. A couple of respondents cited that wearing a mask made them tired.

“*It is not easy to keep it for eight hours per day. Some people say that they have got used to it. I have not*.” (Assistant nurse, public sector)

## Discussion

Our mixed-methods study provides important insights into the both breadth and depth of factors that influence COVID-19-specific IPC practices in LTCFs. Our survey among HCWs identified that domains driving the behaviours were specific to the environmental context and resources (inadequate staffing), reinforcement (management follow-up and feedback), and beliefs about capabilities. Our qualitative study explained how these domains influenced compliance with COVID19-specific IPC practices.

Our study findings align with the growing body of research noting that, although sufficient knowledge is fundamental for effective IPC [17,18], the practices are linked with a number of other behavioural determinants that need to be addressed to improve IPC practices [6,14]. In addition, our study highlights the multifaceted nature of behavioural determinants by showing how TDF domains overlap and blend into one another. Namely, beliefs about capabilities were shown to be linked with knowledge, professional role, and environmental context and resources. This finding is important for understanding the flexible and pluralistic nature of behavioural determinants so as not to evaluate them in a silo and to take them into consideration when planning for interventions to include a mix of techniques and tactics accordingly [19].

Inadequacy of staffing during the pandemic was identified as a factor hindering compliance with IPC practices. One reason was that the altered work responsibilities were often time-consuming, such as staging the mealtimes or scheduling visits for the residents. Time constraints are a common predictor of HCW non-compliance with IPC practices worldwide [20,21] and visitors are a common source of additional workload for HCWs [22]. The use of PPEs and additional cleaning processes have also been reported to increase workload and reduce HCWs’ time and energy to comply with ICP measures during the pandemic [6]. Nevertheless, adequate staffing during emergency situations remains an issue that must be carefully considered.

Our findings also indicate that HCWs in LTCFs appreciate a management culture that provides positive feedback, encourages participation and is physically present. Several studies confirm the importance of management support to encourage IPC behaviours [6]. In addition, leadership prioritisation of IPC has been identified as one of the key actions to implementing appropriate IPC policies among home-based health staff, particularly during a crisis situation such as the COVID-19 pandemic [21].

Face-to-face guidance and follow-up by the management were perceived as important, despite the rapid digitalisation of the world over the past decade. In many countries [23], including Finland, [24] people have become accustomed to using online apps in their everyday lives, even for healthcare workers. They routinely communicate via smartphone apps and use public and the private sector online learning and service platforms. There is a need to further investigate how to improve management culture and feedback. In LTCFs, perhaps management can consider using hybrid strategies that mix face-to-face communication with online communication tools or perhaps management can make the online communication more engaging. They can be trained to use more interactive and participatory tools and methods when communicating with their staff via online applications.

Our findings showed that beliefs about capabilities played an important role in encouraging HCWs’ compliance with IPC measures during the pandemic. This manifested in insufficient knowledge of the nature of the duties, IPC application and in the required infrastructure. In alignment with these findings, previous studies show that HCWs who enjoy a higher level of self-efficacy perceive themselves as more capable of complying with IPC measures [25] and they are more motivated to use them during epidemics [26].

Our study highlighted that HCWs in LTCFs had challenges managing new knowledge during the pandemic. The information was often too much, too generic or changed so often that it was difficult to stay updated. Information was also provided by multiple sources which added to the confusion. Impractical IPC guidance during infectious disease outbreaks has also been reported in other countries such as in the United Kingdom and South Korea due to unstandardised or changing guidance [27,28]. In addition, excess information can reduce the trust of nursing staff towards health authorities and public health responses and therefore demotivate compliance with IPC measures, as was noted in number of studies conducted during the pandemic [29,30]. To address the lack of knowledge, there should be a focus on ensuring that IPC-related information is readily available, clear, consistent and applicable in the context of LTCFs at all times to all HCWs. IPC recommendations should be LTCF-specific rather than generic to ensure that they can be implemented in the context of LTCFs with high-need and high-risk residents including those with cognitive impairments [31]. Communication efforts should be coordinated by only a few sources but should be multifaceted to give more opportunities for busy HCWs to obtain the information [32]. With enhanced knowledge, nursing staff could also identify facility-based approaches to manage new tasks that they have come to perceive as impossible, such as keeping physical distance from the residents or increased hand hygiene measures.

During the past decade, LTCFs in Finland have shifted to a ‘home-like’ environment from an ‘institution-like’ environment, which has reduced the private space for HCWs and has made compliance with some IPC measures, such as hand hygiene or use of PPE, more challenging for the nursing staff. These infrastructural limitations should be also considered in the IPC guidance.

Overall, belief in capabilities can be best enhanced by providing positive coping messages from the management to enhance the confidence of HCWs in IPC practices and to increase their motivation to mitigate the spread of COVID-19 [33].

The acceptance of PPE use did not appear as a strong barrier to IPC implementation in this study, although Finland did experience a lack of PPE at the beginning of the pandemic, and in particular a lack of medical masks as in many other countries [33]. The situation was rectified in autumn 2020.

Our study had some limitations. Firstly, although this study reached across almost all regions in Finland and among different types of healthcare staff in public and private LTCFs, the sample is likely biased towards those facilities that are interested in IPC. Secondly, self-assessment can pose a bias as the psychological mechanisms that underlie bias self-assessment occur below awareness, and accordingly are difficult to address [33]. In addition, there may be a social desirability bias as the interviews were conducted by phone and the interviewer may not have always created a strong rapport with the interviewees where they felt at ease to speak freely. Because of the limited sample size, it was not possible to compare barriers and facilitators between different types of LTCFs or the differences between different types of IPC measures. Our target audience included all staff members of LTCFs. Future research should focus on specific IPC measures such as mask and respiratory use and those who have first-hand experience caring for COVID-19 patients.

## Conclusions

The staff in LTCFs in Finland note several factors that influence their ability and willingness to comply with COVID-19-specific IPC practices during the pandemic, which are tied to staffing, management, knowledge and infrastructural factors. Interventions to improve compliance of IPC must be multifaceted to address the complex and overlapping contextual factors that are driving the behaviours of HCWs in LTCFs.

## Supplementary Data

199000 Supplement
