# Supplementary material for: Exploring behavioural factors influencing COVID-19-specific infection prevention and control measures in Finland: a mixed-methods study, December 2020 to March 2021
Source: Euro Surveill. 2022 Oct 6;27(40):2100915. doi: 10.2807/1560-7917.ES.2022.27.40.2100915 (PMC9540522; doi:10.2807/1560-7917.ES.2022.27.40.2100915)
Supplement: Supplement [file 21-00915_LOHINIVA_SUPPLEMENT.pdf]

Supplementary material

This supplementary material is hosted by Eurosurveillance as supporting information alongside the article " Exploring behavioral factors influencing COVID-19 specific infection prevention and control measures in Finland: a mixed methods study, December 2020 to March 2021", on behalf of the authors, who remain responsible for the accuracy and appropriateness of the content. The same standards for ethics, copyright, attributions, and permissions as for the article apply. Supplements are not edited by Eurosurveillance and the journal is not responsible for the maintenance of any links or email addresses provided therein.

Supplementary file 1: Question guide for qualitative interviews including open-ended and semi-structured questions.

We would like to start this interview by learning about the difficulties that you faced in implementing pandemic-specific IPC measures during the pandemic when caring for patients and the reasons for those difficulties.

Knowledge

Can you tell me about pandemic-related IPC recommendations or policies in your facility?

What do you know about them?

Are there any gaps in what you know about them?

Do you have difficulties obtaining information about them?

Social/ professional role

What is your role in the following pandemic-related IPC measures? And the role of others?

To what extent is the following pandemic-specific IPC recommendation part of your professional role?

How is this influencing your performance at work?

Skills

How easy or difficult would you find acting on pandemic-specific IPC recommendations?

Do you have the skills to follow these recommendations?

Beliefs about capabilities

How confident are you that you can follow pandemic-specific IPC recommendations?

If confidence low: what would make you feel more confident?

How well equipped are you to follow the recommendations?

Beliefs about consequences

What do you think will happen if you follow pandemic-related ICP recommendations?

What do you think are the benefits of doing this, for A. You? B. Patients? C. Pandemic

In your opinion, do the benefits of following these recommendations outweigh the costs?

What do you think happens if you d not follow pandemic-specific IPC measures?

Memory, attention, and decision processes

Is following pandemic-specific IPC recommendations something you usually do?

How will you remember to do this in the future?

Environmental context and resources

What environmental factors or resources help or hinder following pandemic-specific IPC recommendations?

Tell me about systems that are in place to support you if any.

Social influences

Do your colleagues follow pandemic-specific IPC recommendations?

Do your colleagues support you to follow pandemic-specific IPC recommendations?

Does someone pressure you to follow pandemic-specific IPC recommendations?

Reinforcement

How would you describe management's influence on pandemic-specific IPC practices?

Probes; Support, pressure, indifferent attitude, micromanagement etc.

Emotions

How do your feelings at the time (mood, feelings towards the patient, fatigue) affect whether or not you can follow pandemic-related IPC recommendations?

Intentions

What kind of situations or issues prompt you to improve your compliance with pandemic-specific measures?

Goal

What do you think about pandemic-related IPC measures as a goal`?

Probes: What would motivate you to take IPC measures as a professional

Behavioral regulations

Have you made any plans to improve your IPC compliance during the pandemic?

Are there things that help to prompt you to do it?

Optimism

Do you feel optimistic about the implementation of pandemic-specific IPC measures? If yes why? If not, why?

Is there anything else you would like to share with us regarding the implementation of pandemic-related IPC measures?

Thank you for the interview!

Supplementary file 2: Codebook displaying the thematic analysis of the qualitative data to shed light on TDF domains; beliefs in capabilities, environmental context & resources, and reinforcement. The thematic analysis is conducted on three levels; codes, categories, and themes.

Nodes/ codebook

Codes	Category	Theme
**TDF domain: Beliefs in capabilities:**		
Too much information	Overflow of information	Sufficiency of information
Information is coming all the time (too much)	Overflow of information	Sufficiency of information
Information is changing all the time	Overflow of information	Sufficiency of information
Information must not be concise (short)	Understandable information	Sufficiency of information
Information needs to include details	Understandable information	Sufficiency of information
Guidelines need to cover all practices	Understandable information	Sufficiency of information
Information that is well structured	Understandable information	Sufficiency of information
It matters how information is written	Understandable information	Sufficiency of information
Use of abbreviations and technical words makes instructions complex	Understandable information	Sufficiency of information
Using illustrations facilitates reading of information	Understandable information	Sufficiency of information
Information can be too generic to be used	Applicability of the information in the context	Sufficiency of information
Details refer to a specific setting, which makes it difficult to use	Applicability of the information in the context	Sufficiency of information

Codes	Category	Theme
Health facilities are different, which make it difficult to be used	Applicability of the information in the context	Sufficiency of information
Guidance refers to handwashing. But our facility does not have many hand washing basis. We use alcohol rub much more	Applicability of the information in the context	Sufficiency of information
We have patients who walk around , the guidance we have received does not take that into account	Applicability of the information in the context	Sufficiency of information
We don't have staff areas at all. The guidance they provide us does not fit us.	Applicability of the information in the context	Sufficiency of information
We cannot comply with the guidance at all. We have too many patients around	Applicability of the information in the context	Sufficiency of information
Our facility is old. Information does not always consider it.	Applicability of the information in the context	Sufficiency of information
Information flow must be continuous	Availability of information	Sufficiency of information
Often an overflow of information in the beginning of epidemic	Availability of information	Sufficiency of information
Information comes late Friday afternoon when nobody is around to read it	Availability of information	Sufficiency of information
If information is e mailed to staff members, not everyone has the time to read them	Availability of information	Sufficiency of information

Codes	Category	Theme
Instructions that are hanged on the wall are useful as everyone can see them and everyone has access to them	Availability of information	Sufficiency of information
Educational sessions are not practical as nursing staff does not have the time	Availability of information	Sufficiency of information
Provision of information during morning meetings reaches all	Availability of information	Sufficiency of information
Management must be well informed to be able to provide information to their staff members	Availability of information	Sufficiency of information
Management must be interested in IPC measures to transfer the information	Availability of information	Sufficiency of information
When management has indifferent attitude, they don't make an effort to provide information to others	Availability of information	Sufficiency of information
It is not possible to ask residents with memory loss to keep physical distance between them or the nursing staff.	Keeping social distance	Nature of the IPC tasks
Residents don't remember that they should keep physical distance	Keeping social distance	Nature of the IPC tasks
Residents are roaming around freely in the facility	Keeping social distance	Nature of the IPC tasks
Residents need assistance that requires close contact	Keeping social distance	Nature of the IPC tasks

Codes	Category	Theme
between residents and nursing staff		
Nursing is not possible without physical contact	Keeping social distance	Nature of the IPC tasks
We don't have enough space to keep physical distancing	Keeping social distance	Nature of the IPC tasks
It is not possible to keep physical distance between residents	Keeping social distance	Nature of the IPC tasks
Family members do not want to follow visiting rules	Communicating with family members	Nature of the IPC tasks
Family members do not care about visiting rules	Communicating with family members	Nature of the IPC tasks
I don't know how should I convince family members to follow visiting rules	Communicating with family members	Nature of the IPC tasks
I don't feel comfortable talking to family members about visit rules	Communicating with family members	Nature of the IPC tasks
Family members do not listen to nursing staff	Communicating with family members	Nature of the IPC tasks
Negotiation skills are needed	Communicating with family members	Nature of the IPC tasks
Communication with family members is difficult	Communicating with family members	Nature of the IPC tasks
Family members do not respect nursing staff	Communicating with family members	Nature of the IPC tasks
Family members are difficult to manage	Communicating with family members	Nature of the IPC tasks
No changing rooms	Space	Infrastructural support

Codes	Category	Theme
No staff rooms	Space	Infrastructural support
Small workspaces	Space	Infrastructural support
Residents are in the near proximity all the time	Location	Infrastructural support
Residents can show up at any moment	Use of space	Infrastructural support
Few handwashing basins	Use of space	Infrastructural support
Masks create allergic reactions	Products	Infrastructural support
No appropriate place for hand hygiene products	Products	Infrastructural support
Hand hygiene products cannot be places in the facility as residents can mistakenly take them (use them)	Use of space	Infrastructural support
**TDF Domain: Environmental context and resources**		
New duties including taking residents to dining room	Modified procedures	Changes in professional duties
Personalized services for residents to keep physical distancing	Modified procedures	Changes in professional duties
Overseeing that physical distancing is complied	Supervision	Changes in professional duties
Reminding and explaining about the Educating about the importance of physical distancing	Education	Changes in professional duties

Codes	Category	Theme
Using stairs during working day instead of elevators	New procedure	Changes in professional duties
Organizing visiting appointments	New procedure	Changes in professional duties
Overseeing visits of family members	New procedure	Changes in professional duties
Organizing visiting places	New procedure	Changes in professional duties
Cleaning visiting places	Modified procedure	Changes in professional duties
Re-organizing visiting places	Modified procedure	Changes in professional duties
No emergency plans include modified staffing plans	Not available	Lack of staff planning for emergencies
Not enough staff for emergency staffing plans	Not possible	Lack of staff planning for emergencies
Emergency staffing plans create problems	Not possible	Lack of staff planning for emergencies
Dedicated nursing staff for corona patients results to lack of nursing staff for other residents	Negative impact	Lack of staff planning for emergencies
No budget to develop specific emergency staffing plans	Not possible	Lack of staff planning for emergencies

Codes	Category	Theme
Not enough staff to modify any staffing related plans	Not possible	Lack of staff planning for emergencies
No guidance how to develop staff management plans	Not available	Lack of staff planning for emergencies
No instructions to develop staffing plans for emergency situation	Not available	Lack of staff planning for emergencies
**TDF Domain: Reinforcement**		
Positive feedback is a sign of appreciation	Appreciated by the staff	Management culture
Positive comments motivate	Appreciated by the staff	Management culture
Positive guidance is good	Appreciated by the staff	Management culture
Positive discussion motivate	Appreciated by the staff	Management culture
Managers intentions to involve staff in planning encourages to work better	Appreciated by the staff	Management culture
Opportunities to talk with supervisor is empowering	Appreciated by the staff	Management culture
Discussions with manager are not common	Not applied	Management culture
Feedback is not routinely given	Not applied	Management culture
Often feedback is negative	Not applied	Management culture

Codes	Category	Theme
No opportunities to receive feedback	Not applied	Management culture
Management is not available in the facility	Not applied	Physical presence
Managers stay in their own workroom	Not possible	Physical presence
Manager stay at home during pandemic	Not possible	Physical presence
Mangers do not make routinely rounds	Not applied	Management culture
During pandemic managers interact with staff less than in normal times	Not applied	Physical presence
Casual staff is not considered in feedback or comments	Not considered	Management culture
Casual staff is not considered as staff so little contact with managers	Not considered	Management culture
Casual staff receives little supervision	Not considered	Management culture

Supplementary file 3

Ordinary logistic regression and penalised ridge logistic regression, theoretical domain framework and related survey questions associated with the compliance of infection prevention and control measures among healthcare workers in long-term care facilities, Finland, December 2020 (n=422).

Predictor		Ordinary logistic regression			Penalised ridge logistic regression		
Domain	Survey question	OR	95%CI	p value	OR	90%CI	p value
Environmental context and resources	How sufficient do you evaluate staffing to be able to comply with COVID-19-specific IPC measures?	0.55	0.32-0.94	0.027	0.52	0.33-0.82	0.018
Reinforcement and beliefs about capabilities	How do you evaluate management influences your ability to comply with COVID-19-specific IPC measures?<br>How do you consider feedback from management influencing your compliance with COVID-19-specific IPC measures?<br>How much are you able to improve your compliance with COVID-19-specific ICP measures?				1.73	1.00-2.99	0.10

COVID-19, coronavirus disease 19; IPC; infection prevention and control; OR, odds ratio; CI, confidence interval
